# Acetabular Bone Defect Classifications in Revision Total Hip Arthroplasty: A Systematic Review of the Literature

**DOI:** 10.1007/s00402-026-06251-1

**Published:** 2026-02-26

**Authors:** Mattia Loppini, Edoardo Guazzoni, Alberto Bulgarelli, Marco Di Maio, Katia Chiappetta, Guido Grappiolo

**Affiliations:** 1 Humanitas University, Rozzano, Italy; 2 IRCCS Humanitas Research Hospital, Rozzano, Italy; 3 University of Trieste, Trieste, Italy

**Keywords:** Total hip arthroplasty, Revision total hip arthroplasty, Acetabular bone defect classification, Acetabular defect, Acetabular bone loss

## Abstract

**Introduction:**

Acetabular bone defects pose significant challenges during revision total hip arthroplasty (rTHA) due to varying bone quality and quantity. Accurate preoperative evaluation and classification systems are essential to guide surgical planning and ensure stable acetabular fixation. Over the years, several classification systems have been proposed, each emphasizing different variables. The aim of this systematic review was to provide a comprehensive overview of existing classification systems for acetabular bone defects in rTHA.

**Materials and methods:**

A systematic review of the literature was performed to identify all original acetabular bone defect classifications. Studies focusing solely on femoral defects and not-surgery oriented were excluded. The details of each classification system have been reviewed, and a comparison of their inter-observer and intra-observer reliability has been performed.

**Results:**

A total of fifteen classification systems were recognized, published between 1986 and 2024. Variables taken into consideration differ considerably from classification to classification. These include defect location and pattern; the quality of remaining bone stock; the presence of ischial lysis; columns and walls integrity, the presence of pelvic discontinuity; cup loosening and/or migration; and the presence or absence of pain. All but one classification system requires intraoperative evaluation for accurate classification.

**Conclusions:**

While numerous classification systems for acetabular bone defects exist, none is universally accepted. Variability in the parameters assessed and the frequent need for intraoperative evaluation hinder replicability and consistency. A universally accepted, reliable classification framework remains a significant unmet need in the management of acetabular bone defects.

## Introduction

Because of increased life expectancy and ageing of the population, the volume of revision total hip arthroplasty (rTHA) is steadily rising. Between 2005 and 2010, the number of patients undergoing rTHA increased by 23%, and projections suggest a further 31% increase by 2030 [1, 2]. However, longer waiting lists mean delayed access to definitive surgery, with patients presenting to the clinic with more advanced disease, greater functional limitation, and more complex reconstructive needs.

Acetabular reconstruction remains one of the most demanding aspects of rTHA. The extent and pattern of bone loss vary widely between patients, and the quality of the remaining host bone often determines whether stable fixation can be achieved with a hemispherical component, augments, or necessitates cage-based reconstruction. Careful preoperative assessment and surgical planning is therefore essential. In this context, acetabular bone defect classifications are intended to provide a structured description of defect morphology and to support the selection of an appropriate reconstructive strategy.

The first classification of acetabular bone defects was published by Gross et al. [3] in 1986 and included three commonly encountered problems: acetabular protrusion, the shelf defect, and the acetabular defect. Since then, several classification systems have been proposed, each emphasizing different parameters, including defect size and location, qualitative assessment of remaining bone stock, integrity of the acetabular walls and columns, involvement of key anatomic landmarks, radiographic signs of loosening and migration, and, in some systems, clinical symptoms such as pain [4–18]. However, a universal consensus on a single comprehensive and reliable classification system and management algorithm is still lacking. The aim of the present systematic review was to provide a structured overview of published classification systems for acetabular bone defects in the setting of revision THA, highlightning their defining variables and their intended role in guiding reconstruction.

## Methods

A comprehensive search of the US National Library of Medicine (PubMed/MEDLINE), Embase, and the Cochrane Database of Systematic Reviews was performed to identify relevant studies in the available English literature published between 1986 and 2024 describing classification systems for acetabular defects associated with rTHA. The database query was constructed using the following words: “total hip arthroplasty” OR THA OR “total hip replacement” OR THR OR “hip arthroplasty”) AND (revision OR revis* OR “revision arthroplasty” OR rTHA) AND acetabul* AND (“bone loss” OR defect* OR “bone defect*” OR deficien* OR “bone stock”) AND (classif* OR classification OR “classification system” OR “classification scheme” OR grading OR “grading system”). Screening was performed by two independent reviers (EG, AB) with eventual discrepancies solved after discussion with the senior researcher (GG). Titles and abstracts were initially screened, followed by full-text assessment of potentially eligible studies.

**Fig. 1:**
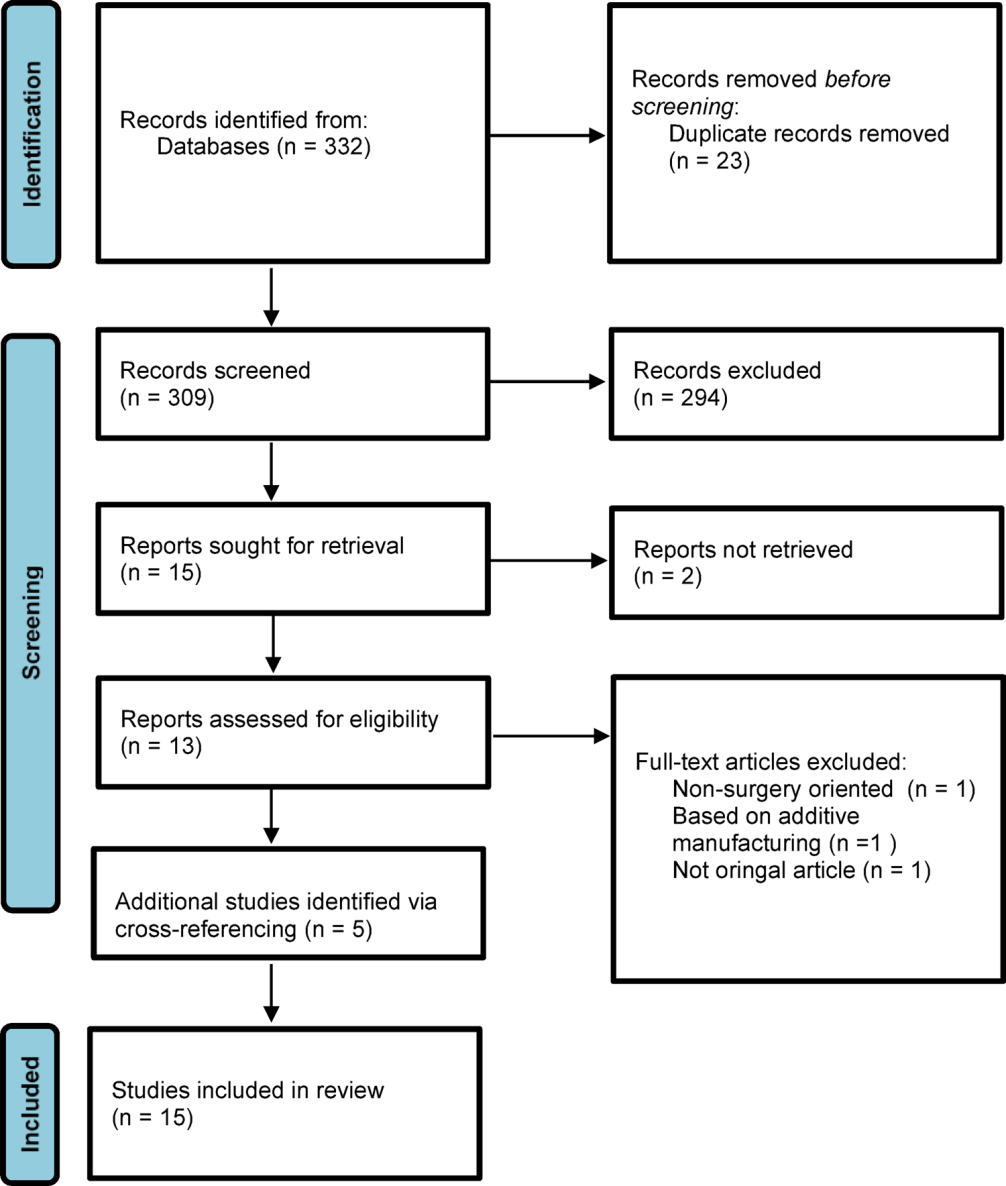
Systematic Review PRISMA (Preferred Reporting Items for Systematic Review and Meta-Analysis) flowchart diagram

All peer-reviewed journals were considered, and all articles reporting classification systems of acetabular bone defects for rTHA were analyzed. Main exclusion criteria were: preclinical studies; classification systems with limited surgical applicability; and non-original articles. While classification systems focused exclusively on femoral bone defects were excluded, classification systems describing both acetabular and femoral defects were reported only for what concerns the acetabulum. Cross-reference research of the selected articles was also performed to identify any additional relevant publications. For each eligible classification, the original article was retrieved, along with studies reporting inter- and intraobserver reliability, where available [expressed with Cohen’s kappa (k) or weighted k (wk)]. A PRISMA (Preferred Reporting Items for Systematic Reviews and Meta-Analyses) flow diagram summarizing the study selection process is presented in Fig. 1.

The level of evidence for each included study was assigned according to the Oxford Centre for Evidence-Based Medicine Levels of Evidence [19]. Methodological quality was assessed using the Coleman Methodology Score as adapted by Sambandan [20]. Each study was scored independently by two reviewers; disagreements were resolved by consensus. Higher scores indicated better methodological quality. Studies were categorized as excellent (85–100), good (70–84), fair (50–69), or poor (< 50).

## Results

The database search returned 332 abstracts. After removal of duplicate articles and the application of inclusion and exclusion criteria, a total of 15 full text reports were sought for retrieval. Of these, however, only 13 were possible to retrieve. Following full-text screening, three studies were excluded. Finally, a total of 15 studies were included in the present systematic review, with 5 studies identified via cross-referencing. All fifteen studies proposed an original classification of acetabular bone defects. Seven [5, 7, 8, 13, 14, 15, 16] of the included studies also addressed femoral bone defects. A synopsis of the key characteristics of included studies is presented in Table 1.

**Table 1:** Classification systems for acetabular bone defects in revision total hip arthroplasty.

Classification	Publication	Study design and level of evidence	Sample characteristics	Defect extension	Defect localization	Mechanical stability	Bone ingrowth	Ischial lysis	Columns integrity	Walls integrity	Pelvic discontinuity	Cup loosening/ migration	Clinical evaluation	Intra-OP Evaluation required	Intra-observer reliability	Inter-observer reliability
Gross	Gross et al. [3] 1985	Case Series Level of evidence IV	44 rTHA Sex: F% 74% Age: 60 (21–84) FU 1.41 (0.5-6)	Yes	\	Yes	\	\	Yes	\	\	\	\	Yes	\	\
Endo-Klinik	Engelbrecht and Heinert [5] 1987	Expert Opinion Level V	\	Yes	\	\	\	\	\	\	Yes	Yes	Yes	Yes	\	\
Gustilo and Paternak	Gustilo and Pasternak [7] 1988	Case Series Level of evidence IV	57 rTHA Sex: F% 37% Age: 59 (25–86) FU 2.8	Yes	\	\	\	\	\	Yes	\	Yes	\	Yes	\	\
Engh	Engh et al. [8] 1988	Case Series Level of evidence IV	160 rTHA Sex: F% 54.3% Age: 55.8 (21–87) FU 4.4 (1.75–7.25)	Yes	\	Yes	Yes	\	\	\	\	\	\	Yes	\	\
Chandler and Penenberg	Chandler [9] 1989	Expert Opinion Level V	\	Yes	\	\	\	\	Yes	Yes	Yes	\	\	Yes	\	\
AAOS	D’Antonio et al. [10] 1989	Expert Opinion Level V	\	Yes	Yes	\	\	\	Yes	\	\	\	\	Yes	k = 0.51(0.37–0.67)	k = 0.24 (0.09–0.41)
Paprosky	Paprosky et al. [11] 1994	Case Series Level of evidence IV	147 rTHA Sex: F% 44.2% Age: 57.4 (24–89) FU 5.7 (2-7.4)	Yes	\	Yes	\	Yes	Yes	Yes	\	Yes	\	Yes	k = 0.45 (0.30–0.62)	k = 0.46 (0.25–0.60)
DGOT	Bettin and Katthagen [13] 1997	Case Series Level of evidence IV	373 rTHA Sex: F% 68% Age 69.5 (31–92)	Yes	Yes	\	\	\	\	\	Yes	Yes	\	Yes	k = 0.36–0.76	k = 0.42–0.76
GIR	Pipino and Molfetta [14] 2000	Expert Opinion Level V	\	Yes	\	\	\	\	\	Yes	\	\	\	Yes	\	\
Saleh	Saleh et al. [15] 2000	Expert Opinion Level V	\	Yes	\	\	\	\	Yes	\	Yes	Yes	\	Yes	wk = 0.87	wk = 0.88
Parry	Parry et al. [16] 2010	Methodological study Level IV	30 rTHA (32 acetabolar) 3 raters	Yes	\	Yes	\	\	\	\	\	\	\	Yes	wk = 0.85	wk CI = 0.35–0.51
Ghanem	Ghanem et al. [17] 2020	Case Series Level of evidence IV	160 rTHA Sex: F% 56.8% Age: 70.5 (45–88)	Yes	\	Yes	\	Yes	\	\	Yes	\	\	Yes	\	\
ADC	Wirtz et al. [18] 2020	Methodological study Level IV	207 rTHA 5 raters	Yes	\	Yes	Yes	\	Yes	\	Yes	\	\	Yes	\	\
SCAR	Walter et al. [22] 2020	Case Series Level of evidence IV	214 rTHA Sex: F% 67.2% Age: 65.1 (29–89) FU 7.2 (0.5–10)	Yes	\	Yes	\	\	\	\	\	\	\	Yes	\	k = 0.94 (95% CI, 0.90–0.98)
Grappiolo and Loppini	Loppini et al. [21] 2024	Methodological study Level IV	101 rTHA 3 raters	Yes	Yes	\	\	Yes	\	\	\	Yes	\	\	k = 0.94 (0.88–0.99)	k = 0.81(0.75–0.87)

Acetabular Defect Classification (ADC); American Academy of Orthopaedic Surgeons (AAOS); Deutsche Gesellschaft für Orthopädie und Traumatologie (DGOT); follow-up (FU); kappa score (k); revision total hip arthroplasty (rTHA); The Italian Society for Revision Arthroplasty (GIR); a stability classification for acetabular reconstruction (SCAR); weighted kappa score (wk)

### Quality assessment

The average quality of evidence according to the Oxford Centre for Evidence-Based Medicine Levels of Evidence was poor. All studies were retrospective except for two [13, 21]. Seven [3, 7, 8, 11, 13, 17, 22] were case series, five [5, 9, 10, 14, 15] were expert opinions, and three [16, 18, 21] were methodological studies.

A total of 1493 patients were included in this review, with all studies having population sizes inferior to 400. All included studies investigated patients with failed total hip arthroplasties requiring revision surgery. However, patient selection was unclear among most studies without distinctions between aseptic and infected etiologies. Only six studies [15, 16, 17, 18, 21, 22] had their statistical methods well-described. The total mean modified Coleman score for the included case series in our systematic review was 28.14 out of 100 (Table 2), ranging from 33 [13] to 20 [17] .

**Table 2:** Quality assessment of case series studies using the modified Coleman Methodology Score

Classification	Publication	CMS
Gross	Gross et al. [3]	24
Endo-Klinik	Engelbrecht and Heinert [5]	NA
Gustilo and Paternak	Gustilo and Pasternak [7]	25
Engh	Engh et al. [8]	32
Chandler and Penenberg	Chandler [9]	NA
AAOS	D’Antonio et al. [10]	NA
Paprosky	Paprosky et al. [11]	30
DGOT	Bettin and Katthagen [13]	33
GIR	Pipino and Molfetta [14]	NA
Saleh	Saleh et al. [15]	NA
Parry	Parry et al. [16]	NA
Ghanem	Ghanem et al. [17]	20
ADC	Wirtz et al. [18]	NA
SCAR	Walter et al. [22]	33
Grappiolo and Loppini	Loppini et al. [21]	NA

Acetabular Defect Classification (ADC); American Academy of Orthopaedic Surgeons (AAOS); a stability classification for acetabular reconstruction (SCAR); Deutsche Gesellschaft für Orthopädie und Traumatologie (DGOT); The Italian Society for Revision Arthroplasty (GIR); not applicable (NA)

### Gross classification (1986)

In 1986, Gross et al. [3] provided one of the earlies descriptions of acetabular defects, identifying three common patterns: acetabular protrusion, the shelf defect, and the acetabular defect. Particular focus was placed on the distinction between structural and non-structural defects depending on weight-bearing capacity. Defect severity was determined by assessing the acetabular rim and the extent of bone loss. Type I defects were defined by minimal or no substantial loss of bone stock. In contrast, type II and type III defects were associated with an uncontained loss of bone stock affecting < 50% and > 50% of the acetabulum, respectively. The initial proposed management was limited to two reconstructive options for all defect types: the allograft and the autograft. However, in 1993 Gross et al. [4] refined these indications (Table 3). While management of type I defects should be guided primarily by the patient’s activity level, for type II and III defects, treatment involves solid grafts and either an uncemented or cemented cup, depending on whether the acetabulum is involved for less or more than 50%, respectively.

**Table 3:** Initial Gross classification (1993)

Type	Description	Management
Protrusion	Contained cavitary defect with intact acetabular walls and columns	Low demand patients: morselized bone and protrusion ring and cemented cup High demand patients: morselized bone and large-diameter uncemented metal-backed cup (porous-coated pressfit held with screws)
Shelf (minor column)	Nonstructural defect with partial loss of the rim and the corresponding acetabular wall and < 50% of the acetabulum	Solid grafts: uncemented cup if <50% of the acetabulum is involved (porous coated pressfit or held with screws); cemented cup if >50% of acetabulum is involved
Acetabular (major column)	Nonstructural defect with one or both column with the corresponding wall defect involving > 50% of the acetabulum	

### Endoklinik classification (1987)

The Endo-Klinik classification [5, 6], also known as the Engelbrecht and Heinert classification, was developed at the Hamburg Endo-Klinik in Germany. It was designed to describe acetabular bone defects encountered in the context of cemented revision THA and is based on both radiographic and clinical findings (e.g., bone resorption, signs of loosening, component migration, and pain). Management varies by grade (Table 4) [5, 6]. Grade I defects are typically managed with a fixed polyethylene flanged cup. For grade II defects, cups with a larger outer diameter and collar are recommended to improve cement pressurization and grouting. Grade III defects are treated similarly, with additional stabilization using screws, pins, or plates if required. Finally, grade IV defects are addressed using a saddle endoprosthesis.

**Table 4:** Endoklinik Classification (1987)

Type	Description	Management
Grade 1 Minimal Bone Loss	Visible resorption, signs of loosening and clinically painful with no prosthesis migration	Fixed cup components made in polyethylene and flanged
Grade 2 Moderate Bone Loss	Increasing resorption, distal migration, presence of a clear lysis zone	Cups with a larger outer diameter and collar for a better cement grouting in the contact zone
Grade 3 Pronounced Bone Loss	Serious loosening with recognizable 3 axis migration	As grade II. If needed, potential stabilization options include screws, pins or plates. Defects should be sealed with cement before reimplanting the components. In case of deep infection, avoid cemented techniques and add an antibiotic.
Grade 4 Severe or Total Bone Loss	Serious loosening with extensive defects and possible pelvic discontinuity	Saddle endoprosthesis

### Gustilo and Pasternak classification (1988)

The Gustilo and Pasternak classification [7] was developed to evaluate acetabular bone loss in the setting of loosening of cemented components (Table 5). Defects are stratified into 4 types according to their extent. Type I denotes minimal enlargement of the acetabular wall with loosening at the cement-prosthesis interface. Type II is characterized by marked enlargement and thinning of the acetabular wall (without wall defects) with loosening at the cement-bone interface. Type III describes localized quadrant wall involvement, whereas Type IV reflects massive global collapse or defects affecting two or more acetabular walls. The authors primarily suggest a cementless revision strategy with bone grafting. Cemented fixation is recommended for selected patients older than 65 years.

**Table 5:** Gustilo and Pasternak Classification (1988)

Type	Description	Management
Type I	Minimal enlargement of the acetabular wall and loosening at the cement-prosthesis interface	Cementless hip arthroplasty If patient > 65 y.o., cemented hip arthroplasty
Type II	Marked enlargement and thinning of acetabular wall (without wall defects) and loosening at the cement-bone interface	Cementless hip arthroplasty with bone grafting
Type III	Local quadrant wall defects: anterior, posterior, superior, and central	
Type IV	Massive and global collapse, or defect involving $\:\ge\:2\:$acetabular walls	

### Engh classification (1988)

The Engh classification [8] provides a qualitative description of acetabular bone loss based on the integrity of the acetabular rim and bed. It describes three patterns: minimal, moderate, and severe damage (Table 6). With minimal damage, the bone is ideally suited for immediate stability and secondary osteointegration. In case of moderate damage, the acetabular rim remains intact and can be used for primary fixation. However, the acetabulr bed may be sclerotic and show medial deficiency, potentially compromising bone ingrowth. Severe damage involves compromise of both the rim and the bed. In this setting, primary fixation can be achieved only by means of structural allografting.

**Table 6:** Engh Classification (1988)

Type	Description	Management
Minimal	Rim and bed intact, bleeding hemispheric surface, Immediate stability and bone ingrowth are possible	Porous coated hemispheric component with 3 spikes
Moderate	Rim intact, bed damaged, mechanical fixation possible, bone ingrowth unlikely	Porous coated hemispheric component with 3 spikes, or smooth-threaded component
Severe	Rim and bed damaged, initial mechanical fixation and bone growth unlikelly	Structural allografting, smooth-threaded component

For reconstruction, Engh proposed two principal implant options: the porous coated hemispheric component with 3 spikes for minimal to moderate defects and a smooth-threaded component for moderate to severe damage due to its ability to provide initial stability in irregular acetabular morphology.

### Chandler and Penenberg classification (1989)

The Chandler and Penenberg classification [9] (Table 7) describes common patterns of acetabular bone loss based on the anatomic landmarks involved. Type I refers to bone loss limited to a portion of the acetabular rim. Type II reflects intra-acetabular bone loss. Type III describes acetabular protrusion with an intact medial wall. However, if the medial wall of the acetabulum is perforated, the defect is classified as Type IV. Finally, combined defects are grouped into a fifth category and subdivided into six progressively more severe subtypes (A–F). Authors proposed allografts as the primary treatment strategy to correct bone loss.

**Table 7:** Chandler and Penenberg Classification (1989)

Type	Description	Management
Type I	Rim defects	Allograft to correct the underline defect
Type II	Intra-acetabular defect	
Type III	Protrusion of the medial wall of the acetabulum	
Type VI	Perforation of the medial wall of the acetabulum	
Type V	Combined acetabular defects:	
	A) Protrusion and perforation of the medial wall of the acetabulum	
	B) Superior rim and intra-acetabular defects	
	C) Superior rim and intra-acetabular defects with medial wall perforation	
	D) Superior acetabular defects and perforation of the medial wall	
	E) Global deficiency (complex anterior, superior, and intra-acetabular area deficiency)	
	F) Column defects ( anterior or posterior rim and intra-acetabular defect with medial wall perforation or pelvic discontinuity)	

### AAOS classification (1989)

**Fig. 2:**
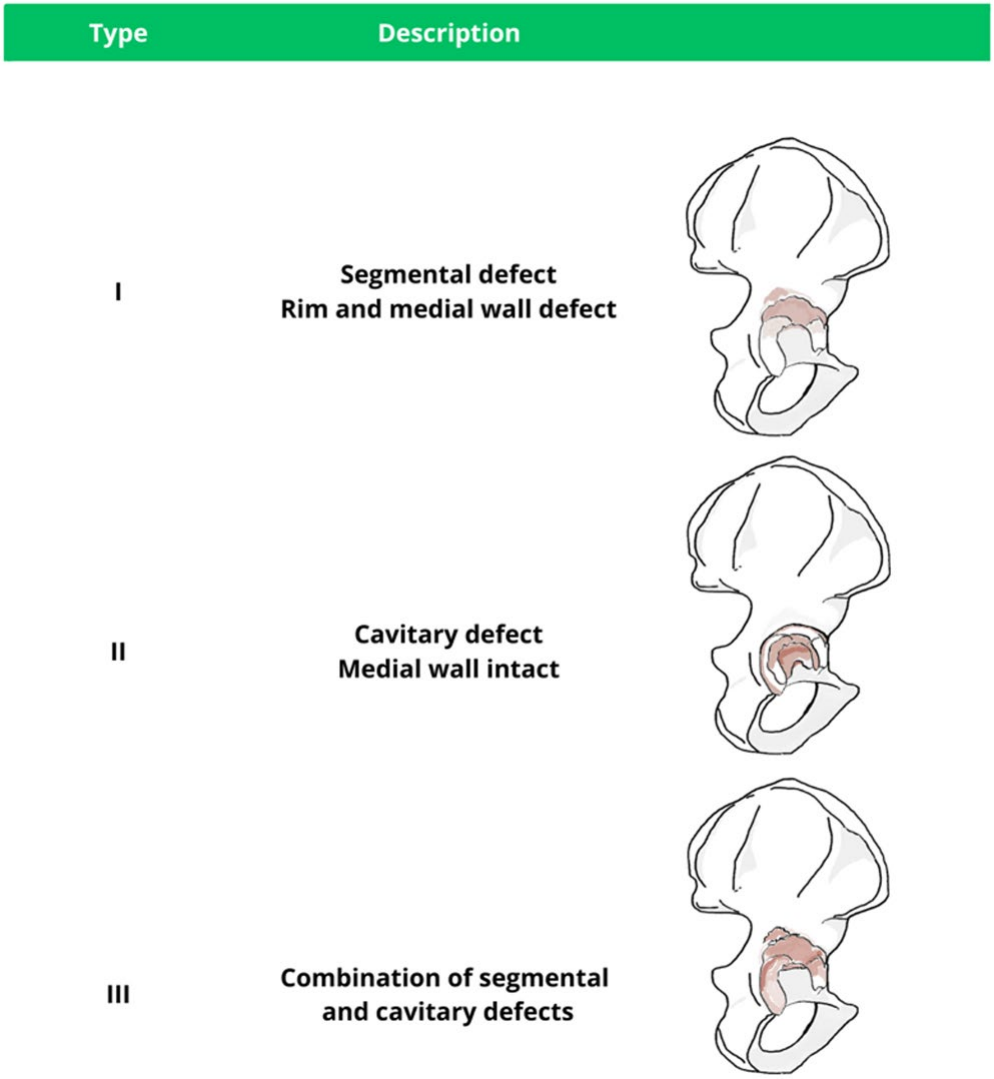
Schematic illustration of the AAOS classification

The American Academy of Orthopaedic Surgeons (AAOS) Classification [10] was developed to provide a comprehensive description of acetabular defects based on their morphology and extent (Table 8). It defines 5 types of defects. The first two are segmental deficiency, referring to complete loss of bone in the supporting hemisphere of the acetabulum, and cavitary deficiency, described as bone loss with an intact acetabular rim. Type III defects represent a combined segmental and cavitary defect (Fig. 2). Lastly, type IV denotes pelvic discontinuity and type V defines arthrodesis.

**Table 8:** The American Academy of Orthopaedic Surgeons (AAOS) Classification (1989)

Type	Description	Management
Type I Segmental Deficiencies	A) Peripheral Superior Anterior Posterior B) Central (medial wall absent)	Grafting techniques (for prosthesis support): block graft (fixed by screws) in situ grafting Avoid bone cement, or particulate grafting (do not give proper support)
Type II Cavitary Deficiencies	A) Peripheral Superior Anterior Posterior B) Central (medial wall absent)	Materials or techniques used for defect filling: Block graft, or Particulate graft, or In situ grafting, or Cement filling (small defects) Prosthesis filling defect
Type III Combined Deficiencies	Multiple defect presenting either as a segmental or cavitary deficiency	First, treat the segmental defect, and onlythen the cavitary deficiency
Type IV Pelvic Discontinuity	Affects both the anterior and the posterior columns with total separation of the superior and inferior acetabulum	Stabilize with plates, bone graft, and screws; it is the first defect to be adressed together with segmental defects
Type V Arthrodesis	Represents a technical deficiency (because the entire bony cavity is filled with bone). The main problem is to find the location of the true acetabulum	Identification and treatment of the true acetabulum with the techniques mentioned for Type IV defects

When multiple patterns coexist, the authors recommend prioritizing treatment of segmental deficiency and pelvic discontinuity, with remaining defects addressed subsequently. Management of type I and II defects is with bone grafting to restore structural support. When present, pelvic discontinuity may be addressed with plates and screws to provide further stability. Finally, in case of arthrodesis, management begins with identification of the true acetabulum, followed by reconstruction tailored to the defect pattern.

### Paprosky classification (1994)

**Fig. 3:**
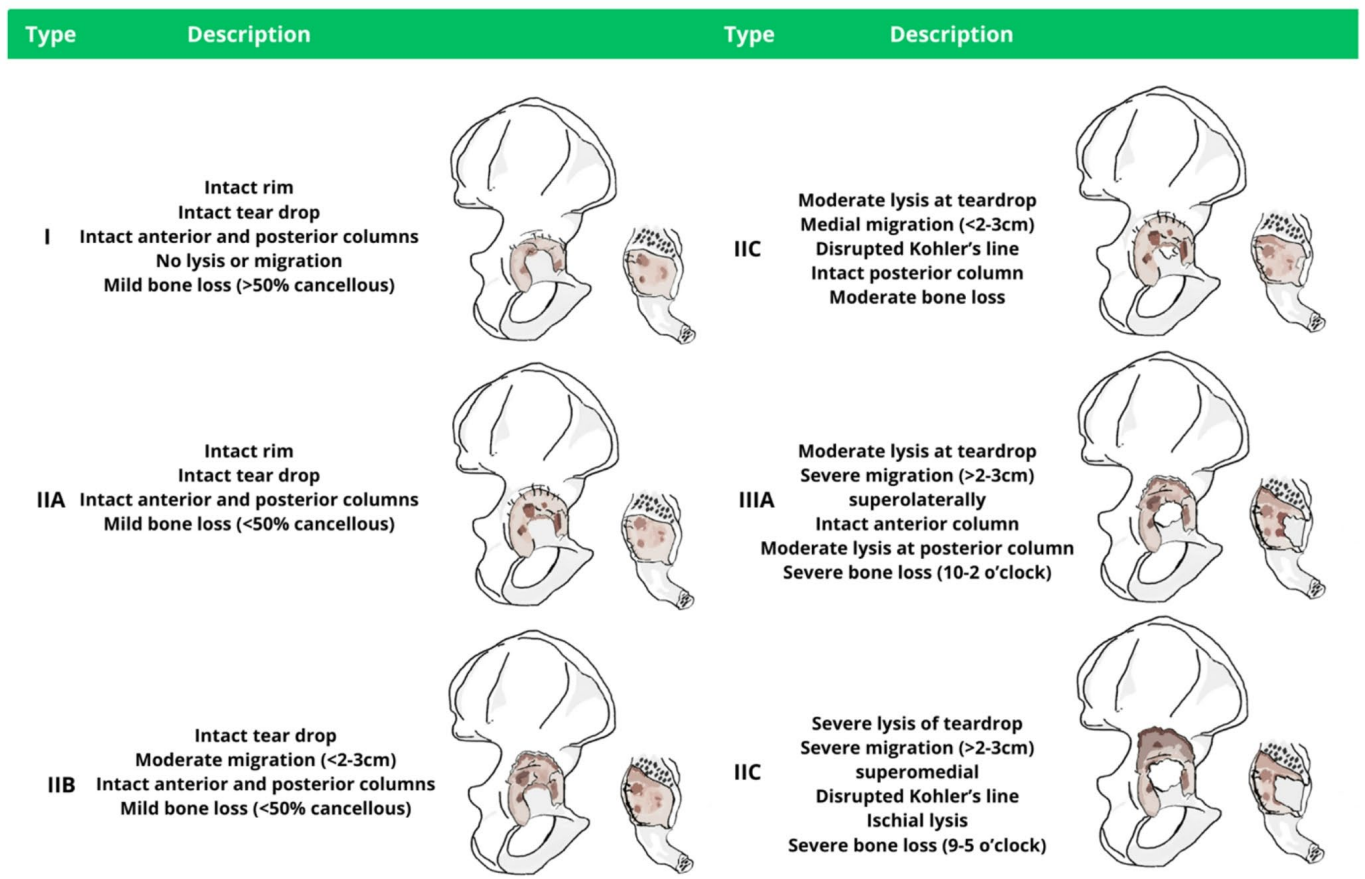
Schematic illustration of the Paprosky classification

The Paprosky classification (Table 9) stratifies acetabular bone loss into three types [11] (Fig. 3). Type 1 defects show minimal focal bone loss with preservation of the hemispheric acetabular shape. The supporting structures are all intact, and there is no hip center (component) migration. Type 2 defects have moderate bone loss, deficient walls but intact columns. The hip center migration is always *<* 2 cm and they are subdivided into A (global cavitation and superior migration), B (superior and lateral migration), and C (deficient medial wall) according to defect location and component migration. Finally, type 3 defects are defined by extensive global erosion with attenuation or destruction of all supporting structures and substantial hip-center migration (> 2 cm). Specifically, in a type 3 A defect, both the acetabular walls and posterior column are destroyed and no longer provide support. The Kohler’s line remains intact, preventing significant medial displacement of the component, but the hip center migrates superolateral (“up and out” deformity). Thirty to 60% of the supporting bone stock is destroyed. Conversely, type 3B defects represent the destruction of all acetabular supporting structures, including both walls and columns, causing the hip center to migrate in a superomedial direction (“up-and-in” deformity). More than 60% of the supporting bone stock is destroyed. Although the original description denotes hip center migration as more or less than 2 cm, a more recent publication liberalizes this migration to less than or greater than 3 cm [12]. The management strategy emphasized by Paprosky is to obtain reliable primary fixation, supplemented by structural restoration when required. In moderate-to-severe defects, the “particulate graft technique” is proposed as a possible reconstructive strategy (i.e., femoral head allograft secured with medullary screws). For type IIIB defects, the authors propose the “arc” graft technique (i.e., a femoral head allograft transected along the coronal plane, so that the natural curve formed by the femoral head, neck, and calcar is in the shape of an arc) secured with reconstruction plates.

**Table 9:** Paprosky classification (1994)

Type	Description	Management
Type 1	Medial wall, anterior and posterior columns are intact. No migration. Remaining bone bed > 50% cancellous.	Porous coated implant, if needed particulate graft
Type 2	Mild to moderate migration <2 cm. Moderate bone loss (remaining bone bed < 50% cancellous). A) Medial wall, anterior and posterior columns are intact. Mild migration (superomedial). B) Medial wall, anterior and posterior columns are intact. Moderate migration (superolateral). C) Moderate lysis of the medial wall, anterior column is disrupted, posterior column is intact. Mild migration (medial).	(A) Large porous coated implant to obtain pressfit (placed “high in position”), or Particulate graft, or Medullary screws to fix femoral head allograft (B) Large porous coated implant to obtain pressfit (placed “high in position”), or Particulate graft, or (C) Number 7 femoral head allograft, with - if needed - cancellous screws Rim pressfit to obtain fixation, and Particulate graft, or Wafer cut femoral head allograft
Type 3	Severe migration > 2 cm Severe bone loss (sclerotic bone bed) A) Moderate lysis of the medial wall, intact anterior column, moderate lysis of posterior column. Severe 10–2 o’clock bone loss. B) Severe lysis of the medial wall disrupted anterior column, severe lysis of posterior column. Severe 9–5 o’clock bone loss.	Proximal tibia or a distal femur 7 shape cut, fixed with cancellous screws; if protrusion defect, treat as 2 C “Arc” graft fixed with reconstruction plates

### Deutsche Gesellschaft für Orthopädie und Traumatologie (DGOT) classification (1997)

The Deutsche Gesellschaft für Orthopädie und Traumatologie (DGOT) classification [13] provides a comprehensive description of acetabular bone loss based on the predonimant defect pattern. Although comprehensive in its morphologic characterization, it does not link defect types to specific reconstructive strategies (Table 10). Type I describes simple cavitary defects with an intact acetabular rim and roof. Type II refers to a unisegmental defect of the acetabular base resulting in perforation, most commonly associated with component protrusion. Type III is a unisegmental roof defect following cranial migration of the loosened cup. Type IV is a unisegmental defect of the front or rear as a result of post-traumatic arthrosis or dysplasia. Type V is a bisegmental defect found in the roof and bottom of the acetabulum, following cranio-central migration of the cup. Type VI is a trisegmental defect found at the roof, bottom, and either the anterior or posterior segment. Finally, type VII indicates pelvic instability.

**Table 10:** Deutsche Gesellschaft für Orthopädie und Traumatologie (DGOT) classification (1997)

Type	Description
Type I Simple cavities	Intact ring structure of the acetabulum, preserved roof and edges of the acetabulum
Type II Unisegmental, base of the acetabulum	Segmental bone defect located in the medial acetabular base causing a perforation
Type III Unisegmental, roof of the acetabulum	Segmental bone defect located in the roof of the acetabulum
Type IV Unisegmental, front or rear acetabular edge	Segmental bone defect located either in the front or in the back of the socket
Type V Bisegmental, acetabular roof and bottom	Defect affecting the acetabular roof and acetabular bottom.
Type VI Trisegmental, acetabular roof, bottom and front or rear	Like type V, plus front or rear acetabular defect
Type VII Pelvic instability	Perforation of the base of the acetabulum, complete pelvic instability; front, rear and bottom acetabular edges are impaired

**Table 11:** Italian Society for Revision Arthroplasty (GIR) classification (2000)

Type	Description	Management
Grade I	Loosening Enlargement and deformation of acetabulum. No wall defect	Filling acetabular cavity (conventional cup, elliptical cup)
Grade II	Loosening Enlargement and deformation of acetabulum. Defect in one wall	Reconstruction of disrupted walls (ring, jumbo cup cemented or cementless) (morselized bone grafts)
Grade III	Loosening Enlargement and deformation of acetabulum. Defect in > 2 walls	Restoring of bone stock (reinforcing ring, stemmed cup) (morselized and impacted or massive allografts)
Grade IV	Massive and overall periacetabular bone loss	Restoring of bone stock Fixation in the intact bone (ring, stemmed cup, segmental reconstructive prosthesis) (morselized or massive bone grafts)

### Italian Society for Revision Arthroplasty (GIR) classification (2000)

The Italian Society for Revision Arthroplasty (GIR), now formally part of the Associazione Italiana Riprotesizzazione (AIR), proposed in 2000 a grading system that links increasing acetabular bone loss to reconstructive strategies (Table 11) [14]. Grade I is defined as the loosening, enlargement, and deformation of the acetabulum without wall defect. Grade II occurs with a defect in one wall in addition to Grade I. Grade III implies impairment of > 2 walls. Grade IV occurs when the periacetabular bone loss is massive. The management of defects is achieved by filling of the defect with the prosthesis, reconstruction of the disrupted walls, restoration of the bone stock, and fixation.

### Saleh classification (2001)

The Saleh classification was developed to provide a valid and reliable classification system for failed hip arthroplasty (Table 12) [15]. Type I describes describes the absence of notable bone stock loss or cup migration, with both columns intact. Type II defects encompass all contained cavitary defects, which may extend beyond the ilioischial line (protrusion). The component may migrate medially or superiorly while the columns remain intact. Type III refers to an uncontained segmental loss of bone stock involving < 50% of the acetabulum. Conversely, type IV refers to uncontained segmental loss exceeding 50% with compromise of both the anterior and posterior columns. Finally, type V represents pelvic discontinuity.

**Table 12:** Saleh classification (2001)

Type	Description	Management
Type I	No notable loss of bone stock, no cup migration and both acetabular columns are largely intact.	Conventional cemented or uncemented cups component
Type II	Contained loss of bone stock, cavitary or volumetric enlargement of the acetabulum. Superior or medial migration of the cup. Intact columns.	Large uncemented cup (high hip center), or Impaction grafting
Type III	Uncontained (segmental) loss of bone stock involving < 50% of the acetabulum, primarily affecting either the anterior or the posterior column.	Large uncemented cup (high hip center) (≥ 50% contact with host bone and will most likely be uncovered superolaterally), or Structural allograft, or Oblong cup
Type IV	Uncontained (segmental) loss of bone stock > 50% of the acetabulum affecting both the anterior and the posterior column. There is no pelvic discontinuity.	Structural allograft with a protective plate or a reconstructive ring (the new implant will have < 50% contact with host bone), or Saddle prosthesis
Type V	Any pelvic discontinuity regardless of the amount of bone loss.	Structural graft, or Reconstruction ring or plate

Management progresses from conventional cemented or cementless components of increasing diameter to more complex solutions for moderate-to-severe defects, including oblong cups, saddle prostheses, plates and rings, and structural allografting.

### Parry classification (2010)

The Parry classification [16] was designed to improve intra- and inter-observer reliability of existing classifications (Table 13). Type A and type B defects have preserved supporting structures but minimal to increasing bone loss, respectively. Finally, type C denotes loss of continuity of supporting structures. Management is aligned with defect severity: type A defects are managed similarly to primary arthroplasty; type B defects typically require bone grafting; and for type C defects, the aim is to convert the lesion into a contained configuration followed by grafting.

**Table 13:** Parry classification (2010)

Type	Description	Management
A	Contained defect with minimal bone stock loss	Treat as primary
B	Contained defect with significant bone stock loss	Graft
C	Uncontained defect	Convert to contained defect and graft

### Ghanem classification (2020)

The Ghanem classification defines four defect types depending on bone quality, defect morphology, and the feasibility to achieve stable fixation [17] (Table 14). In type I defects, the bone is supportive, 3-point fixation is possible, and the acetabulum is hemispherical. While type II defects retain supportive bone but present a cavitary and/or oval defect morphology, type III defects do not allow 3-point fixation. Finally, type IV defects are characterized by pelvic discontinuity.

**Table 14:** Ghanem Classification (2020)

Type	Description	Management
Type I	Possible 3-point fixation within the boundaries of the acetabular wall, hemispherical configuration of the acetabulum	Hemispherical cementless/cemented revision cup ± allogenic cancellous bone
Type II	Possible 3-point fixation within the boundaries of the acetabular wall, cavitary/ oval configuration of the acetabulum	Cementless oval cups or spherical cups with augmentation parts ± allogenic cancellous bone
Type III	Impossible 3-point fixation within the boundaries of the acetabularwall, cavitary configuration of the acetabulum with severe bone loss or pelvic discontinuity	Cementless acetabular cup with cranial strap± iliac stem + allogenic cancellous bone or cup-cage system + allogenic cancellous bone
Type IV	Impossible 3-point fixation within the boundaries of the acetabular wall, pelvic discontinuity with major bone loss and destruction of iliac bone	Custom-made partial pelvic replacement

Recommended management ranges from a hemispherical cementless revision cup for Type I, to oval/spherical revision cups with augments and cancellous allograft for Type II. Type III defects are treated with a cementless cup with adjuncts as required (e.g., cranial strap, iliac stem) and cancellous allograft, or alternatively a cup–cage construct with allograft. Type IV defects are managed with custom-made partial pelvic replacement.

### Acetabular defect classification (ADC) classification (2020)

The ADC classification is based on the integrity of the acetabular rim and supporting structures [18] (Table 15), defining four main defect types of increasing severity with subgroups to specify location.

**Table 15:** Acetabular defect classification (ADC) classification (2020)

Type	Description	Management
Type 1	Contained defects, intact rim and acetabular cancellous deficiency	
	1 A) randomly distributed cancellous defects	Pressfit cup or screw-in cup, and Impaction bone grafting
	1B) lysis of the superomedial aspect	
	1 C) deficiency of the medial wall, which does not affect the anterior or posterior column	Like A/B defects, or cup and cage, or modular cage, and impaction bone grafting
Type 2	Noncontained defect of the acetabular rim ≤ 10 mm and cancellous bone defects	Augment-and-cup, or augment-and-(modular)-cage/oblong, or cup/cranial, or socket system, and impaction grafting
	2 A) defect in the superolateral rim	
	2B) deficient posterior column (horizontal plane)	As type A, plus flanges and/or iliac peg
	2 C) combination of A and B and weight bearing portion of the rim	
Type 3	Noncontained, structural defects over the acetabular rim.	Augment-and-(modular)-cage, and impaction bone grafting
	3 A) involving the superior aspect	
	3B) involving the posterior column	
	3 C) being a combination of both A and B	
Type 4	Pelvic discontinuity	
	4 A) Supportive superior bone stock	Iliac–ischial plating, and augment-and-(modular)-cage, or oblong cup, or cranial socket system with iliac peg and additional flanges, and impaction bone grafting of the medial and superomedial aspect of the acetabulum
	4B) Nonstructural superior rim defect under/equal 10 mm in the vertical plane	Like A, and custom individualized monoblock pelvic replacement with tripolar cup system (dual mobility)
	4 C) Structural superior rim defect over 10 mm	Custom individualized monoblock pelvic replacement with tripolar cup system (dual mobility), and impaction bone grafting of the medial and superomedial aspect of the acetabulum

Type 1 consists of a contained defect with an intact rim and cancellous deficiency: 1A includes scattered lytic areas; 1B involves superomedial lysis; and 1C denotes isolated medial wall deficiency.

Type 2 comprises uncontained, non-structural rim defects measuring ≤ 10 mm in the vertical plane with concomitant cancellous loss: 2A describes a defect affecting the superolateral rim; 2B involves the posterior column on the horizontal plane; and 2C combines A and B, including the weight-bearing portion of the rim.

Type 3 refers to uncontained, non-structural defects of the acetabular rim that exceed 10 mm in the vertical plane. A-C subgroups follow the same structure as Type 2 defects. Finally, type 4 encompasses various pelvic discontinuity with disruption of the bone stock between the ischium and ilium. Subtypes are determined by remaining superior bone stock: 4A indicates superiorly supportive bone, 4B a non-structural superior rim defect ≤ 10 mm, and 4C a structural superior rim defect > 10 mm. Management strategies are determined according to sub-grade (Table 15).

### SCAR classification (2020)

The SCAR classification (i.e. a stability classification for acetabular reconstruction) is an intraoperative, stability-based system developed by Walter et al. (Table 16). Classification relies primarily on the remaining acetabular containment and is determined through a trial-cup stability test performed at the anatomical hip centre (35–40° inclination, 10–15° anteversion) [22]. 

**Table 16:** SCAR Classification (2020)

SCAR type	Stability and key defect features	Typical reconstruction strategy
I	Stable, fully contained acetabulum; intact rim and medial wall	Standard press-fit cup
IIA	Stable; >2/3 rim integrity and > 2/3 containment (defect may be anterior/superior/posterior)	Impaction grafting + standard cup (biological solution)
IIB	Stable; >2/3 rim integrity and > 2/3 containment (defect may be anterior/superior/posterior)	Jumbo cup without grafting (implant-based solution)
IIIA	Unstable; often migration and rim deficiency; typically < 2/3 containment	Revision cup and/or cage + grafting and/or metal augments (combined solution)
IIIB	Unstable; often migration and rim deficiency; typically < 2/3 containment	Special revision cups (e.g., flanged, multi-screw constructs) (monobloc solution)
IV	Unstable Type III pattern with trans-acetabular fracture involving both columns	Monobloc constructs: cages, Burch–Schneider-type devices, or jumbo cups with iliac fixation elements
V	Most severe: unstable, massive deformity, pelvic discontinuity and/or extensive osteolysis	Custom-made implants

SCAR Type I represents a stable, fully contained acetabulum with intact rim and medial wall, that is typically managed with standard press-fit cups. Type II defects remain overall stable with > 2/3 rim integrity and > 2/3 containment. Treatment may be with impaction grafting with a standard cup (IIA - biological solution) or use of a jumbo cup without grafting (IIB - implant-based solution). Type III denotes an unstable situation, often with component migration and rim deficiency, typically with < 2/3 containment. Similarly to type II defects, management may be with to alternative strategies: revision cups and/or cages combined with grafting or metal augments (IIIA - combined solution) or speical revision cups (e.g., flanged, multi-screw constructs) for reconstruction (IIIB – monobloc solution). Type IV defects are comparable to Type III but complicated by a trans-acetabular fracture involving both columns, prompting the use of monobloc constructs such as cages, Burch–Schneider-type devices, or jumbo cups with iliac fixation elements. Finally, type V captures the most severe unstable scenarios—massive deformity, pelvic discontinuity, and/or extensive osteolysis—typically requiring custom-made implants. The reported inter-observer reliability of the SCAR was 0.94 (95% CI, 0.90–0.98).

### Grappiolo Loppini classification (2024)

The classification of acetabular defects put forward by Grappiolo and Loppini in 2024 [21] is designed to allow surgeons to pre-operatively categorize the extent of bone loss using a standard AP pelvis radiograph. This classification helps guide further radiological evaluation and implant selection, enabling precise planning for reconstruction and the choice of the most suitable implant components to ensure long-term implant survival.

**Fig. 4:**
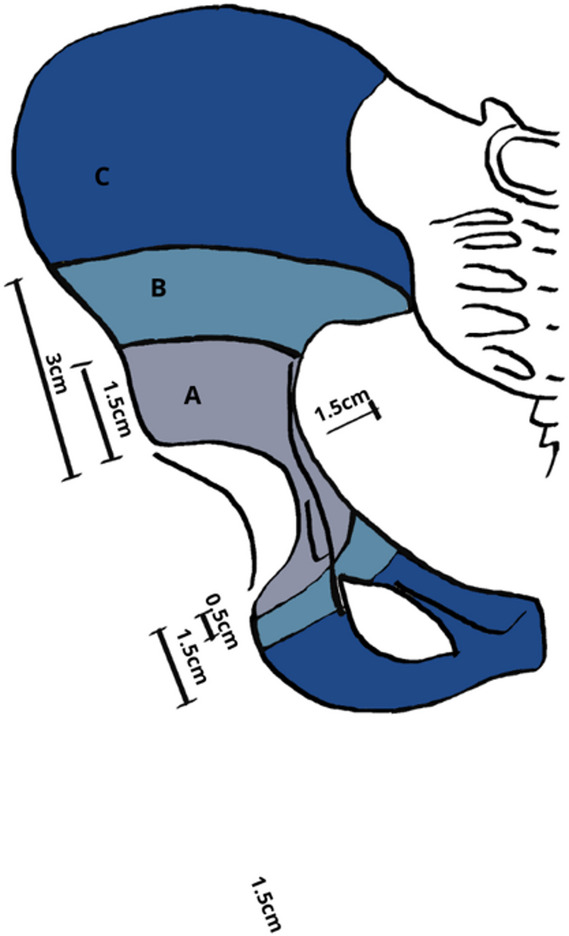
Schematic illustration of the Grappiolo Loppini classification

To assess the extent of the defect, the authors used a line drawn parallel to the one passing through the teardrops. The periacetabular region was classified into three zones based on the extension of the bone defect (Fig. 4). Zone A was defined as an acetabular bone defect located between 1.5 cm from the superior acetabular rim and 0.5 cm from the teardrop inferiorly, without crossing of the ilioischial (Kohler) line [7]. Zone B was defined as a defect that extended beyond Zone A but remained within 3 cm of the acetabular rim, within 1.5 cm of the teardrop inferiorly, and within 1.5 cm medial to the ilioischial line. Zone C included all defects reaching beyond Zone B. (Table 17). Treatment recommendations were tiered by zone. The proposed treatment for Zone A defects is the use of uncemented hemispherical cups with or without the use of screws without further imaging studies. For zone B defects, recommended options include a jumbo cup (i.e., an acetabular shell > 62 mm for females and > 66 mm for males) [8], a hemispherical cup with a single augment, or bone impaction grafting (BIG) with either a cemented or uncemented hemispherical cup. A computed tomography scan (CT)scan may be performed case-by-case, depending on the surgeon’s experience. For zone C defects, the authors advised more complex reconstructions such as custom implants, a triflange cage, or modular strategies (e.g., hemispherical cup with ≥ 2 augments, cup–cage, cup-on-cup, BIG with an anti-protrusio cage). In this setting, pelvic CT is strongly recommended, with a 3D-printed reconstruction when available. Using this framework, treatment prediction accuracy was 88.1% (k weighted: 0.68) for the fellow, 86.2% (k weighted: 0.61) for the experienced orthopedic surgeon, and 87.6% (k weighted: 0.65) for the resident, resulting in an average of 87.3% (k weighted: 0.65). The inter-rater reliability was 90.1% (k: 0.81 with a 95% CI of 0.75–0.87), while the intra-rater reliability for the two evaluations conducted by the fellow one month apart was 97.5% (k: 0.94 with a 95% CI of 0.88–0.99).

**Table 17:** Grappiolo Loppini classification (2024)

Zone	Description	Management
A	Superiorly: 1.5 cm from acetabular roof Medially: no invasion of Kohler line Inferiorly: 0.5 cm from tear drop	As primary THA
B	Superiorly: 3 cm from acetabular roof Medially: 0–1.5 cm from Kohler line Inferiorly: 1.5 cm below the tear drop	Hemispherical cup + single augment, jumbo cup, bone impaction grafting with a cemented or uncemented hemispherical cup
C	Superiorly: > 3 cm Medially: > 1.5 from Kohler line inferiorly: > 1.5 cm from tear drop	Custom implant, triflange cage, modular reconstructions (hemispherical cup + > 2 augment, cup-and-cage, cup-on-cup), BIG with the use of an antiprotrusio cage

## Discussion

Over the years, several classification systems have been proposed to describe the morphology of acetabular bone defects and to support decision-making in rTHA. Each framework emphasizes specific variables, and even when similar parameters are taken into consideration, they are defined and assessed using different criteria. As a result, a direct comparison between classification systems of acetabular bone defects is challenging.

While size defect and extension is consistently addressed across all classifications, a structured appraisal of its location is present only in the AAOS, DGOT, and Grappiolo Loppini classification [10, 13, 21]. The quality of the remaining bone stock was variably described in terms of supportive or non-supportive for the new implant in the Gross, Engh, Paprosky, Parry, Ghanem, and ADC classifications [3, 4, 8, 11, 16, 17, 18]. Notably, the Engh classification additionally considers the likelihood of bone ingrowth, while the ADC explicitly accounts for cancellous deficiency [8, 18]. The assessment of ischial lysis represents another critical issue influencing the reconstruction, as ischial integrity is necessary when cage implantation is contemplated. Among previous classifications, this aspect was taken into account only by the Paprosky, ADC, and Grappiolo and Loppini classification systems [11, 18, 21]. 

Assessment of acetabular walls and columns is essential for defining defect severity and achievable fixation and represents a central theme across most classifications. Gross, AAOS, Saleh, and ADC considered columns impairment [3, 4, 10, 15, 18], Gustilo and Paternak, and GIR evaluated wall enlargement [7, 14]. Chandler and Penenberg and Paprosky took into consideration both variables [9, 11].

Pelvic discontinuity is a sign of advanced bone loss and predicts increasing complexity of reconstruction. It is mentioned by the Endo-Klinik, Chandler and Penenberg, DGOT, Saleh, Ghanem and ADC classifications [8, 9, 13, 15, 17, 18]. Cup loosening and/or migration are evaluated in the Endo-Klinik, Gustilo and Paternak, Paprosky DGOT, Saleh and Grappiolo and Loppini classifications [5, 7, 11, 13, 15, 21]. In contrast, clinical symptoms are rarely integrated. Notably, pain is explicitly considered only in the Endo-Klinik classification [5]. The SCAR classification was the only classification system derived from an intraoperative trial-cup stability test [22]. 

An ideal classification system should be easy to apply and, importantly, reliable. However, formal analysis of inter- and intra-observer agreement has been performed for only a limited number of systems, namely the AAOS, Paprosky, DGOT, Saleh, Parry, SCAR, and Grappiolo Loppini classifications [10, 11, 13, 15, 16, 21, 22]. In the available studies, the AAOS, Paprosky and DGOT classifications reported mean kappa values below 0.80 for both intra- and inter-observer reliability. Only, the SCAR and Grappiolo Loppini classifications achieved kappa values above 0.80 for intra- and inter-observer relialibiliy [21], indicating “strong” agreement according to the criteria proposed by McHugh M.L. et al. [23]. The Parry and Saleh classifications reported only weighted kappa (kw), precluding a direct comparison with studies using an unweighted kappa.

With the exception of the Grappiolo Loppini classification [21], all reported classifications require an intraoperative assessment for precise definition of the defect. As such, it may be useful as a screening tool to flag complex cases that warrant further radiological evaluation. In their classification, the authors highlight three-dimensional (3D) computed tomography (CT) reconstruction and 3D-printed models as practical tools for a more detailed preoperative workup in rTHA. By providing a clearer depiction of volumetric bone loss and periacetabular bone quality, these modalities may enhance the surgeon’s ability to anticipate intraoperative findings and plan reconstruction accordingly. Moreover, 3D-printed models allow to test whether the remaining bone stock supports fixation with a standard acetabular cup or whether a patient-specific implant should be considered [24]. However, a standardized CT-based defect analysis is not without limitations, including higher costs and higher radiation dose compared to conventional radiographic examination [25]. In addition, they do not provide the possibility of an automated defect analysis. The process of segmentation has to be manually controlled and adjusted if necessary. Again, the quality of the final 3D-reconstruction and defect analysis are highly dependent on the experience of the user, who is, in most cases, an engineer and not a surgeon or a radiologist. Therefore, the critical final steps of validating the reconstruction and integrating the findings into a surgical plan ultimately remain the responsibility of the operating surgeon [25].

Although pooling all available acetabular defect classifications, this study is not without limitations. Its findings were limited by the methodological quality of included studies, their poor quality of evidence and small samples sizes. Seven out of fifteen studies were case series, five were expert opinions, and three were methodological studies. Future studies should evaluate the reliability and validity of acetabular bone defect classification systems using larger cohorts and more rigorous study designs and how these change across different imaging modalities.

## Conclusions

Several classification systems have been developed to evaluate acetabular bone loss, yet no single classification has been accepted as the gold-standard. Variability in the parameters assessed and the frequent need for intraoperative evaluation hinder replicability and consistency. While all available classifications consider defect size and extension, each describes acetabular bone defects focusing on a combination of specific variables. These include defect location and pattern; the quality of remaining bone stock; the presence of ischial lysis; columns and walls integrity; the presence of pelvic discontinuity; cup loosening and/or migration; and the presence or absence of pain. Future studies should evaluate the reliability and validity of acetabular bone defect classification systems using larger cohorts and more rigorous study designs and how these change across different imaging modalities.

## Data Availability

No datasets were generated or analysed during the current study.
